# Unsupervised Insulator Defect Detection Method Based on Masked Autoencoder

**DOI:** 10.3390/s25144271

**Published:** 2025-07-09

**Authors:** Yanying Song, Wei Xiong

**Affiliations:** 1 Detroit Green Technology Institute, Hubei University of Technology, Wuhan 430068, China; 2211621225@hbut.edu.cn; 2 School of Electrical and Electronic Engineering, Hubei University of Technology, Wuhan 430068, China

**Keywords:** insulator defect detection, unsupervised learning, masked autoencoder (MAE), vision transformer (ViT), random masking training, interval masking inference, defect detection and localization

## Abstract

With the rapid expansion of high-speed rail infrastructure, maintaining the structural integrity of insulators is critical to operational safety. However, conventional defect detection techniques typically rely on extensive labeled datasets, struggle with class imbalance, and often fail to capture large-scale structural anomalies. In this paper, we present an unsupervised insulator defect detection framework based on a masked autoencoder (MAE) architecture. Built upon a vision transformer (ViT), the model employs an asymmetric encoder-decoder structure and leverages a high-ratio random masking scheme during training to facilitate robust representation learning. At inference, a dual-pass interval masking strategy enhances defect localization accuracy. Benchmark experiments across multiple datasets demonstrate that our method delivers competitive image- and pixel-level performance while significantly reducing computational overhead compared to existing ViT-based approaches. By enabling high-precision defect detection through image reconstruction without requiring manual annotations, this approach offers a scalable and efficient solution for real-time industrial inspection under limited supervision.

## 1. Introduction

Insulators are critical components of electrified railway systems, ensuring mechanical support for overhead conductors and electrical insulation along transmission lines. Over extended periods of service, these devices are subjected to harsh environmental conditions, including extreme temperatures, precipitation, sandstorms, and mechanical stress, which can induce a range of defects such as breakage, surface contamination, cracking, and partial shielding [1]. If not promptly identified and mitigated, such degradations pose serious risks to the safety and reliability of railway operations.

Current methods for insulator defect detection fall into two main categories: traditional techniques based on handcrafted features, and supervised deep learning approaches. Classical methods typically exploit edge, texture, or keypoint descriptors in conjunction with shallow classifiers, but their performance is constrained by limited prior knowledge and poor generalization to complex defect morphologies [2]. Supervised deep learning models offer improved detection accuracy through end-to-end training on annotated datasets [3,4], yet their deployment in industrial settings is often hindered by the scarcity of defect samples, high labeling cost, and pronounced class imbalance [2]. Unsupervised learning has recently gained attention as a viable alternative, leveraging the distribution of normal data to detect anomalous deviations [5]. However, existing unsupervised methods, particularly those based on convolutional autoencoders, frequently exhibit limited capacity for capturing global context and localizing fine-grained defects [6].

To overcome persistent challenges in insulator defect detection, including the scarcity of annotated defect samples, pronounced class imbalance, and the computational demands of high-resolution image analysis, we introduce an unsupervised detection framework based on a masked autoencoder (MAE) [7]. The proposed architecture builds upon the vision transformer (ViT) [8], which has demonstrated superior performance in capturing long-range dependencies through its self-attention mechanism. Unlike convolutional neural networks (CNNs), which are inherently biased toward local features, ViT processes images as sequences of patches and models global context more effectively, an essential capability for detecting structural anomalies in complex insulator environments. This property makes ViT particularly suitable for unsupervised learning tasks, where robust and generalizable representation learning from defect-free samples is critical.

This study introduces several key innovations that advance unsupervised insulator defect detection:

(1) A high-ratio random masking scheme (e.g., 75%) is employed during training to suppress image redundancy, enabling the model to concentrate on learning essential representations from defect-free samples. This approach mitigates the dependence on extensive labeled datasets and addresses practical constraints such as limited data availability, labor-intensive annotation, and severe class imbalance.

(2) A dual-interval masking reconstruction fusion strategy is proposed for inference. By interchanging masked regions between two reconstruction passes and integrating the outputs, the model is discouraged from replicating defect regions present in the input. This mechanism enhances reconstruction discrepancy in defective areas, improving sensitivity to small-scale anomalies and robustness to large-scale structural defects.

The rest of this paper is organized as follows. Section 2 reviews related research on visual self-supervised learning, MAE architectures, and state space modeling. Section 3 provides a detailed description of the proposed unsupervised defect detection method based on ViT-MAE. Section 4 describes the dataset preparation, experimental settings, evaluation metrics, and presents a comprehensive analysis of the results. Finally, Section 5 summarizes the main findings, discusses current limitations, and outlines possible directions for future research.

## 2. Related Work

Advances in image recognition have provided new tools for insulator defect detection, yet early approaches remain rooted in handcrafted feature extraction combined with shallow classifiers. Traditional methods rely on manually engineered visual descriptors: edge detectors such as Canny [9] and Sobel [10] are used to capture surface contours, while texture-based techniques, including local binary patterns (LBP) [11] and Gabor filters, extract repetitive structures along insulator sheds. Feature matching under geometric variation is typically handled by keypoint-based descriptors such as SIFT [12] and SURF [13]. These features are then classified using shallow models, most commonly support vector machines or random forests [14], via statistical learning paradigms. For example, LBP features can be employed to identify anomalous textures, followed by SVM-based classification to distinguish defective from intact regions. However, the expressiveness of handcrafted features is inherently limited by the assumptions and prior knowledge encoded by the designer. As a result, such methods often fail under complex conditions, including novel defect morphologies, sudden illumination changes, or contamination artifacts, leading to elevated false negative rates and reduced robustness in real-world applications.

The advent of deep learning has enabled supervised approaches to surpass the limitations of traditional methods through end-to-end feature learning. Convolutional neural networks, such as ResNet [15] and DenseNet [16], extract hierarchical representations from low-level edges to high-level semantics via stacked convolutional layers. Object detection frameworks, including YOLO [17] and Faster R-CNN [18], facilitate simultaneous defect localization and classification, while semantic segmentation models such as U-Net [19] and DeepLab [20] allow for pixel-level defect annotation. In the context of insulator inspection, models like CACNN [21] enhance detection accuracy through multi-scale feature fusion, whereas RCCAEN [22] leverages an autoencoder architecture to jointly reconstruct and classify abnormal regions. Supported by large-scale annotated datasets, supervised models effectively capture the semantic characteristics of defects, achieving detection accuracies exceeding 95% in complex scenarios involving cracks, contamination, and structural anomalies. However, real-world deployment remains hindered by practical constraints: defect samples are scarce, often constituting less than 5% of available data; annotation requires domain-specific expertise and is labor-intensive; and the pronounced class imbalance between defective and normal instances undermines generalization, particularly for rare or novel defect types.

To avoid reliance on annotated defect samples in supervised learning, unsupervised approaches aim to model the distribution of normal data and identify anomalies as deviations from learned patterns. Early efforts in this domain have primarily employed autoencoder-based architectures. Standard autoencoders reconstruct input images through an encoder–decoder pipeline, with defects inferred from localized reconstruction errors. However, CNN-based autoencoders rely on local receptive fields, limiting their ability to model the global structure of insulators and increasing the likelihood of missed detection in cases of large-area damage. Variational autoencoders incorporate probabilistic modeling to capture the latent distribution of normal features, yet often produce overly smooth reconstructions, thereby reducing localization accuracy [23]. Denoising autoencoders improve robustness by reconstructing clean images from corrupted inputs [24], but similarly remain constrained by their dependence on local features, impairing sensitivity to subtle anomalies such as micro-cracks.

Recent efforts have also explored the utility of Transformer-based models for infrastructure monitoring or anomaly detection tasks. For example, Inadomi and Chun [25] propose a spatially aware graph-based Transformer for predicting bridge deterioration, demonstrating the applicability of attention mechanisms in modeling structural dependencies in civil systems. Yang and Guo [26] propose an unsupervised anomaly detection model based on the ViT architecture, which integrates a memory module to record normal sample features, thereby offsetting the anomaly reconstruction problem and strengthening feature representation. Lee and Kang [27] propose a ViT-based model for unsupervised anomaly detection and localization, which aims to reflect normal information by additionally learning global relationships between image patches. These methods leverage the global context modeling capability of Transformers, but often rely on dense self-attention over full-resolution images, resulting in substantial computational cost. In contrast, our approach utilizes a masked autoencoder with high-ratio sparse random masking and an asymmetric encoder–decoder structure, which reduces computation while maintaining high detection performance. Moreover, unlike prior work, which primarily evaluates on generic industrial benchmarks (e.g., MVTec AD [28]), our method is tailored for the safety-critical domain of catenary insulator inspection, which has received comparatively limited attention in the literature.

## 3. Proposed Method

**Figure 1:**
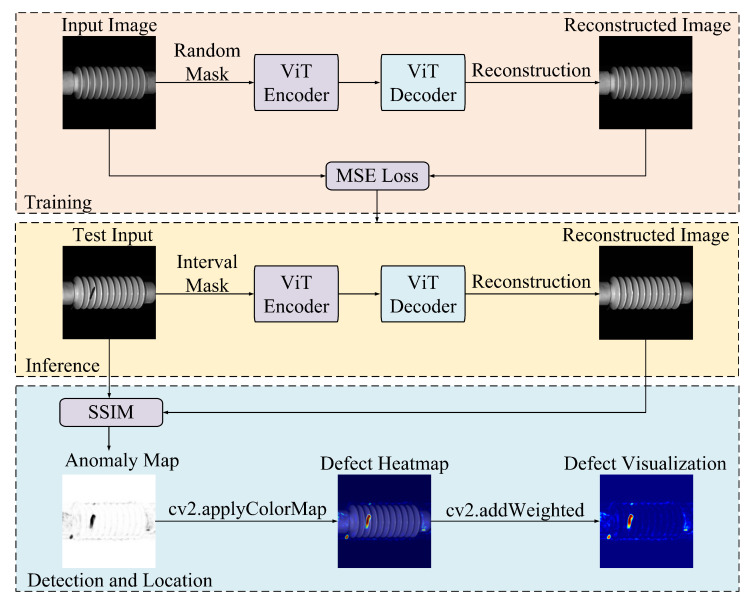
The proposed ViT-MAE model for unsupervised insulator defect detection.

To address the challenges posed by limited defect samples, severe class imbalance, and the high computational cost of processing high-resolution images, we propose an unsupervised defect detection method based on the masked autoencoder (MAE) framework, as shown in  Figure 1. The approach employs a vision transformer (ViT) backbone to enable efficient and scalable visual representation learning. During training, a high-ratio random masking strategy is applied to defect-free insulator images, allowing the model to learn robust, generalizable features without reliance on annotated defect data. At inference, an interval masking strategy is introduced to guide image reconstruction. Defect regions are identified by computing the structural similarity index (SSIM) [29] between the original and reconstructed images, yielding pixel-level anomaly scores for accurate detection and localization.

### 3.1. Random Masking Training

**Figure 2:**
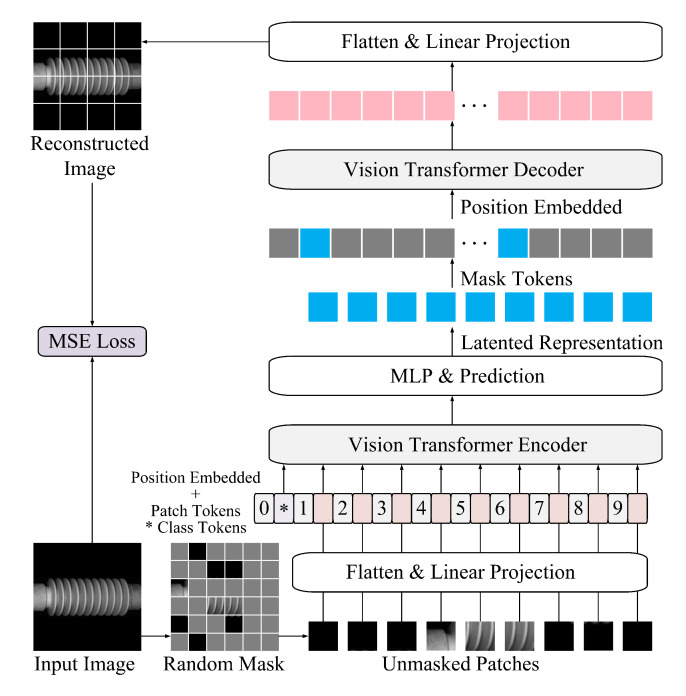
The architecture diagram of the training phase.

The MAE-based training procedure is illustrated in  Figure 2. A non-symmetric encoder–decoder architecture is adopted, where the encoder operates exclusively on unmasked image patches, while a lightweight decoder reconstructs the original image using both the latent representations and placeholders corresponding to the masked patches.

Each input image is first resized to 224×224 pixels and partitioned into non-overlapping patches of 16×16 pixels, yielding a total of 14×14 grid patches. A random masking strategy is then applied, with 75% of the patches masked uniformly at random. This high masking ratio reduces input redundancy and forces the model to infer missing content from limited visible context, thereby encouraging the learning of robust, high-level representations.

The encoder, based on the ViT architecture, processes only the visible patches. Each patch is linearly projected into a fixed-dimensional token and augmented with learnable positional embeddings to retain spatial structure. These tokens are then passed through a stack of Transformer blocks, producing latent features that encode global semantic information. Notably, masked patches are excluded from the encoding phase, allowing the encoder to focus exclusively on informative content, thus improving both model accuracy and computational efficiency.

The decoder receives the latent representations from the encoder, along with placeholder tokens for the masked patches. Positional embeddings are reintroduced before passing the sequence through a shallower and narrower stack of Transformer blocks. This lightweight design reduces computational burden and accelerates convergence, while maintaining sufficient capacity for accurate image reconstruction. Such architectural asymmetry is particularly suited for large-scale insulator datasets, where efficiency is paramount.

The output of the decoder is mapped back to pixel space via a linear projection layer, generating a reconstructed version of the original image. The reconstruction quality is measured using the mean squared error (MSE) between the reconstructed and original pixel values. This loss serves as the training objective, guiding the model to optimize its reconstruction fidelity and, by extension, its representation learning capabilities.

### 3.2. Interval Masking Inference

**Figure 3:**
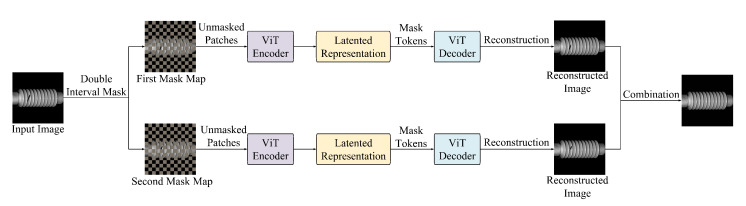
The architecture diagram of the inference phase.

The inference procedure is illustrated in  Figure 3. To enhance the robustness and fidelity of anomaly detection, an interval masking strategy is introduced, where two complementary masking patterns are applied to the input image. Each masked variant is independently reconstructed using the pretrained MAE, and the final output is generated by fusing the reconstructions.

Consistent with the training phase, the input image is first divided into non-overlapping patches of size 16×16 pixels. In the first reconstruction pass, an interval-based masking pattern is applied; for instance, masking patches are located at all odd-indexed rows and columns. This masking scheme is agnostic to defect locations, thereby simulating a spatially uniform occlusion. The masked image is then processed by the MAE’s asymmetric encoder–decoder architecture to generate a partial reconstruction.

In the second pass, the masking pattern is reversed: patches previously visible are now masked, and vice versa. This complementary masking ensures that all regions of the image are reconstructed at least once in a masked state, enabling the model to infer missing content based solely on learned normal patterns.

The final reconstructed image is assembled by selectively retaining only those patches reconstructed from their masked states in each pass. In other words, for each spatial location, only the synthesized output generated while that patch was masked is preserved, while outputs from unmasked (i.e., directly propagated) patches are discarded. This fusion mechanism prevents the model from unintentionally copying defects from the input into the output, a known issue when unmasked patches, which may contain anomalies, are passed through the decoder without modification.

By relying exclusively on the model’s reconstructions of masked regions, where no direct access to original pixel values is available, the fused image more faithfully reflects the structure expected in defect-free regions. The resulting discrepancy between the input and fused reconstruction is thereby amplified in defective areas, facilitating more accurate and stable anomaly detection.

This strategy proves particularly effective in two critical aspects: it enhances sensitivity to subtle, small-scale anomalies such as micro-cracks, and it disrupts the continuity of large-scale structural defects, thereby reducing the likelihood that the model will erroneously reconstruct such regions as normal. Together, these advantages substantially improve the reliability and precision of unsupervised insulator defect detection.

### 3.3. Defect Detection and Localization

**Figure 4:**
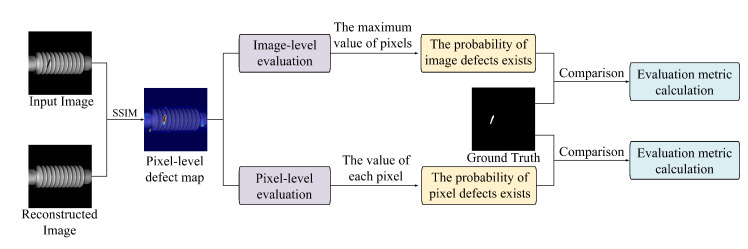
The architecture diagram of the test phase.

The defect detection and localization procedure is depicted in  Figure 4. This phase utilizes the structural similarity index (SSIM) between test images and their corresponding reconstructions to generate pixel-level anomaly maps. These maps are subsequently used for both pixel-level and image-level evaluation, allowing quantitative assessment of defect presence based on ground-truth labels.

The test image $P_{t}$ is first processed through the pretrained MAE to yield a reconstructed image $P_{r}$. Since the model is trained exclusively on defect-free images, it captures the distribution of normal visual patterns. During inference, a spaced interval masking strategy guides the reconstruction, ensuring that all regions of the test image are subjected to masked reconstruction at least once.

To quantify differences between $P_{t}$ and $P_{r}$, the SSIM is computed at each pixel location using the standard formulation:

(1) $$\mathrm{SSIM}\left(P_{t},P_{r}\right)=\frac{\left(2\mu_{t}\mu_{r}+C_{1}\right)\left(2\sigma_{tr}+C_{2}\right)}{\left(\mu_{t}^{2}+\mu_{r}^{2}+C_{1}\right)\left(\sigma_{t}^{2}+\sigma_{r}^{2}+C_{2}\right)}$$

where $\mu_{t}$ and $\mu_{r}$ represent the local means of the test and reconstructed images, $\sigma_{t}$ and $\sigma_{r}$ their variances, $\sigma_{tr}$ their covariance, $C_{1}$ and $C_{2}$ are stability constants. Applying this formula across the entire image produces a pixel-level anomaly map, in which lower SSIM values indicate greater deviation from the learned normal pattern and thus higher probability of a defect.

To enhance perceptual sensitivity, we adopt the multi-scale SSIM (MSSIM) formulation [30], which averages SSIM values over progressively coarser image resolutions. A Gaussian-weighted kernel is applied in each local window to align statistical weighting with human visual perception and reduce block effects caused by discrete patch processing.

The resulting anomaly map represents the probability of each pixel belonging to a defective region. Pixels with SSIM scores closer to zero are more likely to correspond to defects, while those approaching one are considered structurally similar to normal content. This pixel-wise probability distribution is then aggregated into a visual defect map, enabling precise localization of anomalies.

For improved interpretability, the anomaly map is transformed into a heatmap using the cv2.COLORMAP_JET color scheme. After scaling the anomaly scores to the [0, 255] range and converting them to the uint8 format, color mapping is applied: blue hues indicate regions of high similarity (normal), while red hues highlight low similarity (anomalous) regions. The heatmap is then overlaid onto the original image using weighted blending via OpenCV’s cv2.addWeighted, preserving structural detail while visually emphasizing defect locations. This composite visualization offers intuitive feedback to human inspectors, facilitating rapid assessment of defect extent and location.

To evaluate performance at the image level, the maximum pixel value in the anomaly map is used as the representative anomaly score for the entire image. This approach assumes that the most deviant region dominates the image-level defect judgment. A high maximum value suggests the presence of a pronounced anomaly, while a low value indicates likely normalcy. These image-level scores are aggregated to construct a global anomaly score map, with values ranging from 0 to 1.

## 4. Experiment and Analysis

### 4.1. Dataset

**Figure 5:**
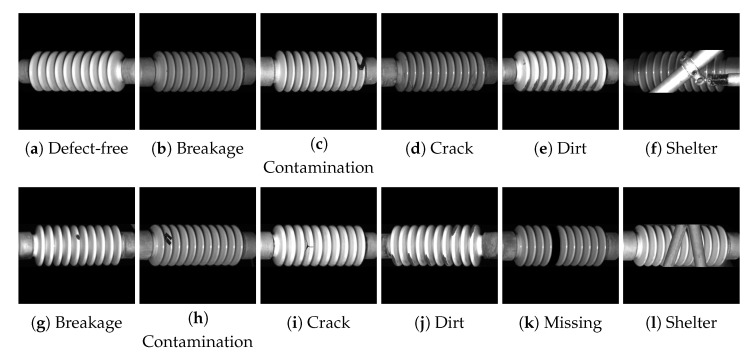
Representative examples from the CID dataset showing different defect categories, such as breakage, contamination, crack, dirt, missing parts, and sheltering. Top row: real defect images (Test_Real); bottom row: simulated defect images (Test_Sim). All images are resized to 224×224 for model input.

The catenary insulator defect (CID) dataset used in this study comprises a training set of 3900 real, defect-free images and a test set containing 393 images, as detailed in Table 1. The dataset is tailored for unsupervised learning, where only normal samples are used during training. The test set incorporates both real and simulated defects and is divided into two subsets, Test_Real (160 images) and Test_Sim (233 images), providing a diverse benchmark for evaluating detection performance under varying conditions. Figure 5 shows representative images of each defect type from the Test_Real and Test_Sim subsets. These diverse and challenging patterns reflect typical field conditions in a high-speed railway environment and are essential for evaluating the generalization capability of unsupervised methods.

To enhance the quality and consistency of the training data, we implement a rigorous data cleaning and preprocessing procedure. Although the training set is officially annotated as defect-free, minor structural anomalies introduced during image acquisition or labeling may compromise the performance of unsupervised models that rely on accurately learning normal distributions. To mitigate this risk, a two-stage cleaning strategy is adopted.

In the first stage, we apply a similarity-based filtering method [31] to exclude images with structural inconsistencies. Two visually representative defect-free images with different orientations are selected as reference templates. Cosine similarity is computed between each training image and the templates. Images exhibiting a similarity score below 0.85 are discarded, as they are more likely to contain noise or latent defects. In the second stage, Sobel edge detection is used to identify and remove images with incomplete contours or blurred edge structures. After both steps, 3550 high-fidelity images are retained to form the final cleaned training set.

Subsequent preprocessing standardizes all retained images to a resolution of 224×224 pixels, with pixel values normalized to a range of [0, 1]. To correct for illumination variability, histogram equalization or contrast-limited adaptive histogram equalization (CLAHE) [32] is applied, thereby improving robustness to changes in lighting conditions and enhancing edge clarity.

To further augment the model’s resilience to localized anomalies, we optionally utilize the CutPaste data augmentation technique [33]. This involves randomly cropping patches from defect-free images, applying geometric transformations (e.g., rotation or translation), and pasting them back into the original image. The resulting pseudo-defect instances introduce structural perturbations while preserving overall visual realism, facilitating robust representation learning in the absence of labeled defects.

For the test sets, we manually review and filter out samples with severe occlusion, corruption, or defect regions smaller than 500 pixels, which may hinder reliable anomaly localization. Furthermore, the test data is statistically validated to ensure a balanced distribution across six defect categories—breakage, contamination, cracks, dirt, missing components, and sheltering—capturing a broad spectrum of real-world fault scenarios.

While our current preprocessing strategy operates in the spatial domain, frequency-domain approaches such as those explored by Rosso et al. [34], who compared CNN-based tunnel monitoring with and without 2D Fourier transforms, may offer complementary benefits. Integrating frequency-aware representations with our reconstruction-based framework is a promising direction for future investigation.

### 4.2. Evaluation Metrics

To comprehensively assess the performance of our proposed insulator defect detection framework, we adopt a dual-level evaluation strategy, reporting both image-level and pixel-level metrics in accordance with established practices in anomaly detection. The primary metrics include the area under the receiver operating characteristic curve (AUROC), precision, recall, and F1-score.

AUROC provides a threshold-independent measure of the model’s ability to distinguish between normal and defective samples. Higher AUROC values indicate greater discriminatory power, reflecting robust performance across a wide range of decision thresholds.

Precision quantifies the proportion of true positives among all samples predicted as positive, capturing the accuracy of defect identification without penalizing missed detection. In contrast, recall (or sensitivity) measures the proportion of true positives correctly identified from all actual defect cases, emphasizing the model’s ability to detect relevant anomalies.

The F1-score, defined as the harmonic mean of precision and recall, offers a balanced assessment by simultaneously accounting for false positives and false negatives. This metric is particularly informative in scenarios with pronounced class imbalance, as is typical in industrial defect datasets.

All reported performance metrics are computed on the designated test set, which comprises the Test_Real and Test_Sim subsets. No validation or calibration set with labeled defects is used during training or threshold selection, in keeping with the unsupervised setting. Thresholds for image-level and pixel-level detection are determined directly on the test data using the anomaly scores generated by the model.

### 4.3. Implementation Details

All experiments are performed on a workstation equipped with an NVIDIA GeForce RTX 3090 GPU (24 GB VRAM), ensuring sufficient computational capacity for large-scale training and evaluation.

The model is trained for 2000 epochs with a batch size of 32, using the Adam optimizer and an initial learning rate of 0.0005. During training, a random masking strategy is employed, where 75% of image patches are masked to encourage the model to learn robust global representations. The loss function is defined as MSE between the reconstructed and original images in pixel space.

At inference, the masking strategy is modified to an interval-based scheme, enabling complementary reconstructions of each test image. Structural discrepancies between original and reconstructed images are then quantified using SSIM, which serves as the basis for defect detection and pixel-level localization.

### 4.4. Comparison with State-of-the-Arts (SOTAs)

Experiments are conducted independently on the Test_Real and Test_Sim subsets to evaluate the performance of our proposed method across both real and simulated defect scenarios. Three established unsupervised anomaly detection methods are reimplemented under identical experimental conditions. STFPM [35] utilizes a student–teacher feature pyramid matching approach; PaDiM [36] employs pre-trained ResNet features combined with Gaussian modeling; and RDAD [37] integrates adversarial training with U-Net-based reconstruction. All comparative methods adopt the same image resolution (224×224), input normalization, and training hyperparameters as the proposed approach to ensure fair and reproducible performance comparisons.

Table 2 presents a comprehensive comparison of image-level defect detection and pixel-level defect localization performance, evaluated separately on the Test_Real and Test_Sim datasets. Across both image- and pixel-level tasks, the proposed ViT-MAE method consistently achieves strong performance, outperforming or closely matching state-of-the-art counterparts.

At the image level, all methods achieve high AUROC values (>96%), indicating the feasibility of unsupervised learning for insulator defect detection. Among the baselines, PaDiM and RDAD perform competitively, with AUROC values exceeding 99% on both subsets. However, the proposed ViT-MAE variants, particularly those incorporating a predetection module (denoted as “w pre”), exhibit consistently superior performance. Specifically, the predetection-enhanced model achieves the highest AUROC (99.66 on Test_Real and 99.64 on Test_Sim), coupled with high precision (99.54/99.26) and recall (99.25/99.22), resulting in near-perfect F1-scores (both 0.99). The comparison between “w pre” and “w/o pre” demonstrates that isolating the insulator region prior to anomaly reconstruction marginally improves overall image-level discrimination.

Pixel-level localization, which demands finer structural sensitivity, presents greater variability across methods. Traditional approaches such as STFPM and PaDiM yield moderate F1-scores (0.47–0.57), reflecting a limited ability to localize small or complex defect regions. In contrast, the proposed ViT-MAE substantially improves localization accuracy, particularly when combined with the predetection step. The “w pre” model achieves F1-scores of 0.65 on Test_Real and 0.70 on Test_Sim, outperforming all baselines. This improvement is attributed to the model’s ability to focus reconstruction exclusively on the insulator body, thereby reducing background interference and enhancing anomaly contrast. Interestingly, the difference in performance between “w pre” and “w/o pre” is more pronounced at the pixel level, suggesting that predetection plays a crucial role in improving spatial precision.

**Figure 6:**
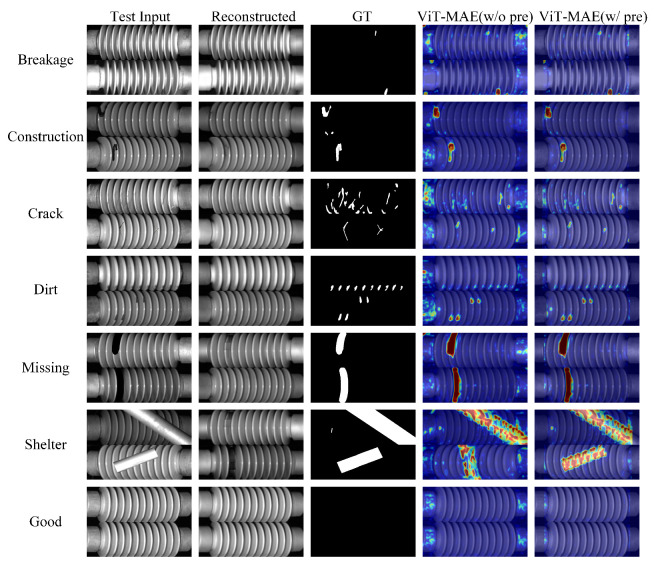
Visualization results of the proposed insulator defect detection and localization method.

Figure 6 shows representative qualitative visualization results of defect detection and localization for various defect types, facilitating visual comparison between different methods.

## 5. Conclusions

This study presents an unsupervised defect detection framework for insulators based on MAE and ViT architectures, offering a principled solution to critical challenges such as annotation scarcity, severe class imbalance, and the structural complexity of real-world defects. The use of high-ratio random masking during training facilitates efficient global representation learning from normal samples, while a dual-interval masking strategy at inference prevents defect information leakage and enhances the precision of reconstruction-based localization. Defect regions are further delineated through pixel-level anomaly scoring via structural similarity metrics, enabling fine-grained and interpretable detection.

Extensive experiments on the public CID benchmark dataset demonstrate the superiority of our proposed method, which achieves AUROC values exceeding 99.6% for image-level detection and F1-score up to 0.70 for pixel-level localization, substantially outperforming state-of-the-art unsupervised counterparts. The adoption of a lightweight decoder ensures low computational overhead without compromising reconstruction fidelity, supporting deployment in time-sensitive industrial scenarios. Taken together, these findings underscore the practical efficacy of ViT-MAE-based architectures in unsupervised visual inspection and provide a promising solution for reliable insulator monitoring in high-speed railway systems.

In future work, we plan to explore hybrid representations that incorporate both spatial and frequency-domain features (e.g., via 2D Fourier or wavelet transforms) to enhance sensitivity to periodic or texture-level anomalies. This direction may complement the MAE’s spatial reconstruction capabilities and benefit defect types such as contamination or surface dirt.

## Figures and Tables

**Table 1:** The statistical overview of the catenary insulator defect (CID) dataset.

		Training Set	Test Set	
			Test_Real	Test_Sim
Defect-free		3900	33	0
Defect	Breakage	0	26	34
	Contamination	0	5	55
	Crack	0	2	58
	Dirt	0	54	6
	Missing	0	0	60
	Shelter	0	40	20
Total		3900	160	233

**Table 2:** Quantitative image-level results for defect detection and pixel-level results for defect localization. (Entries denoted as A/B correspond to performance on the Test_Real and Test_Sim datasets, respectively).

	Methods	AUROC	Precision	Recall	F1-Score
**Image-level Detection**	STFPM [35]	96.68/98.30	93.98/97.06	98.43/99.14	0.96/0.98
	PaDiM [36]	99.49/99.59	99.20/99.14	97.64/98.71	0.98/**0.99**
	RDAD [37]	99.12/**99.68**	96.18/98.72	99.21/**99.57**	0.98/**0.99**
	ViT-MAE (w/o pre)	99.60/99.62	99.44/98.46	98.89/99.21	**0.99**/**0.99**
	ViT-MAE (w pre)	**99.66**/99.64	**99.54**/**99.26**	**99.25**/99.22	**0.99**/**0.99**
**Pixel-level Localization**	STFPM [35]	91.40/97.37	40.54/47.99	69.54/63.81	0.47/0.52
	PaDiM [36]	92.45/98.06	42.71/51.92	68.08/69.97	0.49/0.57
	RDAD [37]	95.58/98.36	46.96/59.43	69.29/**74.08**	0.52/0.64
	ViT-MAE (w/o pre)	96.90/97.69	59.67/54.70	74.83/70.58	**0.65**/**0.70**
	ViT-MAE (w pre)	**96.93**/**98.68**	**60.25**/**60.10**	**74.89**/70.80	**0.65**/**0.70**

## Data Availability

Due to privacy restrictions, the benchmark dataset used in this study for detecting catenary insulator defects is not currently available to the public.

## References

[1] Zhang T., Zhong S., Xu W., Yan L., Zou X. (2024). Catenary Insulator Defect Detection: A Dataset and an Unsupervised Baseline. IEEE Trans. Instrum. Meas..

[2] Deng F., Luo W., Wei B., Zuo Y., Zeng H., He Y. (2022). A Novel Insulator Defect Detection Scheme Based on Deep Convolutional Auto-Encoder for Small Negative Samples. High Volt..

[3] Gu Z., Wang Y., Xue X., Wang S., Cheng Y., Du X., Dai P. Railway Insulator Defect Detection with Deep Convolutional Neural Networks. Proceedings of the 12th International Conference on Digital Image Processing (ICDIP 2020).

[4] Zhang Z., Huang S., Li Y., Li H., Hao H. (2022). Image Detection of Insulator Defects Based on Morphological Processing and Deep Learning. Energies.

[5] Getachew Shiferaw T., Yao L. (2024). Autoencoder-Based Unsupervised Surface Defect Detection Using Two-Stage Training. J. Imaging.

[6] Bergmann P., Löwe S., Fauser M., Sattlegger D., Steger C. Improving Unsupervised Defect Segmentation by Applying Structural Similarity to Autoencoders. Proceedings of the 14th International Conference on Computer Vision Theory and Applications (VISIGRAPP 2019).

[7] He K., Chen X., Xie S., Li Y., Dollár P., Girshick R. Masked Autoencoders Are Scalable Vision Learners. Proceedings of the IEEE/CVF Conference on Computer Vision and Pattern Recognition (CVPR 2022).

[8] Dosovitskiy A., Beyer L., Kolesnikov A., Weissenborn D., Zhai X., Unterthiner T., Dehghani M., Minderer M., Heigold G., Gelly S. An Image is Worth 16x16 Words: Transformers for Image Recognition at Scale. Proceedings of the 9th International Conference on Learning Representations (ICLR 2021).

[9] Canny J. (1986). A Computational Approach to Edge Detection. IEEE Trans. Pattern Anal. Mach. Intell..

[10] Amer G.M.H., Abu Shaala A. Edge Detection Methods. Proceedings of the 2nd World Symposium on Web Applications and Networking (WSWAN 2015).

[11] Ojala T., Pietikäinen M., Harwood D. (1996). A Comparative Study of Texture Measures with Classification Based on Featured Distributions. Pattern Recognit..

[12] Lowe D.G. (2004). Distinctive Image Features from Scale-Invariant Keypoints. Int. J. Comput. Vis..

[13] Bay H., Tuytelaars T., Van Gool L. SURF: Speeded Up Robust Features. Proceedings of the European Conference on Computer Vision (ECCV 2006).

[14] Breiman L. (2001). Random Forests. Mach. Learn..

[15] He K., Zhang X., Ren S., Sun J. Deep Residual Learning for Image Recognition. Proceedings of the IEEE Conference on Computer Vision and Pattern Recognition (CVPR 2016).

[16] Huang G., Liu Z., Van Der Maaten L., Weinberger K.Q. Densely Connected Convolutional Networks. Proceedings of the IEEE Conference on Computer Vision and Pattern Recognition (CVPR 2017).

[17] Redmon J., Divvala S., Girshick R., Farhadi A. You Only Look Once: Unified, Real-Time Object Detection. Proceedings of the IEEE Conference on Computer Vision and Pattern Recognition (CVPR 2016).

[18] Ren S., He K., Girshick R., Sun J. (2017). Faster R-CNN: Towards Real-Time Object Detection with Region Proposal Networks. IEEE Trans. Pattern Anal. Mach. Intell..

[19] Ronneberger O., Fischer P., Brox T. U-Net: Convolutional Networks for Biomedical Image Segmentation. Proceedings of the Medical Image Computing and Computer-Assisted Intervention (MICCAI 2015).

[20] Chen L.C., Papandreou G., Kokkinos I., Murphy K., Yuille A.L. (2018). DeepLab: Semantic Image Segmentation with Deep Convolutional Nets, Atrous Convolution, and Fully Connected CRFs. IEEE Trans. Pattern Anal. Mach. Intell..

[21] Tao X., Zhang D., Wang Z., Liu X., Zhang H., Xu D. (2020). Detection of Power Line Insulator Defects Using Aerial Images Analyzed With Convolutional Neural Networks. IEEE Trans. Syst. Man Cybern. Syst..

[22] Liu W., Liu Z., Wang H., Han Z. (2020). An Automated Defect Detection Approach for Catenary Rod-Insulator Textured Surfaces Using Unsupervised Learning. IEEE Trans. Instrum. Meas..

[23] Kingma D.P., Welling M. (2013). Auto-Encoding Variational Bayes. arXiv.

[24] Vincent P., Larochelle H., Bengio Y., Manzagol P.A. Extracting and Composing Robust Features with Denoising Autoencoders. Proceedings of the 25th International Conference on Machine Learning (ICML 2008).

[25] Inadomi S., Chun P.j. (2025). Spatially Aware Markov Chain-Based Deterioration Prediction of Bridge Components Using a Graph Transformer. Comput.-Aided Civ. Infrastruct. Eng..

[26] Yang Q., Guo R. (2024). An Unsupervised Method for Industrial Image Anomaly Detection with Vision Transformer-Based Autoencoder. Sensors.

[27] Lee Y., Kang P. (2022). AnoViT: Unsupervised Anomaly Detection and Localization with Vision Transformer-Based Encoder-Decoder. IEEE Access.

[28] Bergmann P., Fauser M., Sattlegger D., Steger C. MVTec AD–A Comprehensive Real-World Dataset for Unsupervised Anomaly Detection. Proceedings of the IEEE/CVF Conference on Computer Vision and Pattern Recognition (CVPR 2019).

[29] Wang Z., Bovik A.C., Sheikh H.R., Simoncelli E.P. (2004). Image Quality Assessment: From Error Visibility to Structural Similarity. IEEE Trans. Image Process..

[30] Wang Z., Simoncelli E.P., Bovik A.C. Multiscale Structural Similarity for Image Quality Assessment. Proceedings of the 37th Asilomar Conference on Signals, Systems and Computers (Asilomar Conf. SSC 2003).

[31] Islam M., Zunair H., Mohammed N. (2024). CosSIF: Cosine Similarity-based Image Filtering to Overcome Low Inter-class Variation in Synthetic Medical Image Datasets. Comput. Biol. Med..

[32] Gonzalez R.C., Woods R.E. (2018). Digital Image Processing.

[33] Li C.L., Sohn K., Yoon J., Pfister T. CutPaste: Self-Supervised Learning for Anomaly Detection and Localization. Proceedings of the IEEE/CVF Conference on Computer Vision and Pattern Recognition (CVPR 2021).

[34] Rosso M., Aloisio A., Randazzo V., Tanzi L., Cirrincione G., Marano G. (2023). Comparative Deep Learning Studies for Indirect Tunnel Monitoring with and without Fourier Pre-processing. Integr. Comput.-Aided Eng..

[35] Wang G., Han S., Ding E., Huang D. Student-Teacher Feature Pyramid Matching for Anomaly Detection. Proceedings of the 32nd British Machine Vision Conference (BMVC 2021).

[36] Defard T., Setkov A., Loesch A., Audigier R. PaDiM: A Patch Distribution Modeling Framework for Anomaly Detection and Localization. Proceedings of the 25th International Conference on Pattern Recognition Workshops (ICPR 2020).

[37] Tien T.D., Nguyen A.T., Tran N.H., Huy T.D., Duong S.T., Nguyen C.D.T., Truong S.Q.H. Revisiting Reverse Distillation for Anomaly Detection. Proceedings of the IEEE/CVF Conference on Computer Vision and Pattern Recognition (CVPR 2023).

